# Relationship between cannabis use and schizophrenia

**DOI:** 10.1192/j.eurpsy.2022.1191

**Published:** 2022-09-01

**Authors:** J.J. Vazquez Vazquez, M.M. Gutiérrez Rodríguez, M.D.L.A. Corral Y Alonso, C. Moreno Menguiano, F. Garcia Sánchez

**Affiliations:** 1 Centro de Salud Mental de Móstoles / Hospital Universitario de Móstoles, Psychiatry, Móstoles, Spain; 2 HOSPITAL UNIVERSITARIO DE MÓSTOLES, Psychiatry, MÓSTOLES (MADRID), Spain

**Keywords:** Cannabis, relationship, psicosis, schizophrénia

## Abstract

**Introduction:**

Numerous studies have shown evidence that cannabis use increases the appearance of psychotic symptoms and disorders, and worsens the course of the disease in those with schizophrenia. However, a causal relationship between cannabis and schizophrenia has not been well established yet.

**Objectives:**

In this presentation we try to review the relationship between cannabis use and prevalence of schizophrenia.

**Methods:**

We performed a search of Medline looking for systematic reviews and methodologically robust studies in the field published in English in the last 5 years.

**Results:**

A number of studies, both cross-sectional and prospective, find a prevalence of schizophrenia several times higher among cannabis users than in non-users. This association becomes stronger the lower the age of cannabis use onset, the higher the amount consumed and the higher the THC concentration are. Half of the patients with a cannabis-induced psychotic disorder turn into a diagnosis of schizophrenia within a few years. So far, it has not been possible to demonstrate a global increased prevalence of schizophrenia in relation to the increase of cannabis use in the population in recent decades.

**Conclusions:**

Cannabis and schizophrenia have a complex relationship model; we still cannot clearly establish whether it is causal or the first works as a trigger for pathology in vulnerable subjects.

**Disclosure:**

No significant relationships.

